# Baclofen and the Alcohol Withdrawal Syndrome-A Short Review

**DOI:** 10.3389/fpsyt.2018.00773

**Published:** 2019-01-22

**Authors:** Gary Cooney, Mathis Heydtmann, Iain D. Smith

**Affiliations:** ^1^Florence Street Mental Health Resource Centre, Glasgow, United Kingdom; ^2^Department of Gastroenterology, Royal Alexandra Hospital, Paisley, United Kingdom; ^3^Kershaw Unit, Gartnavel Royal Hospital, Glasgow, United Kingdom

**Keywords:** baclofen, alcohol withdrawal, alcohol dependence, alcohol history, review

## Abstract

The Alcohol Withdrawal Syndrome (AWS), which may occur with or without delirium, is a frequent consequence of sudden alcohol cessation in patients with moderate to severe Alcohol Dependence Syndrome (ADS). Withdrawal as a result of habituation to alcohol is part of the definition of the Alcohol Dependence Syndrome (ICD10). Since the recognition of Delirium Tremens, in the early nineteenth century, the management of the syndrome, an acute medical emergency, has proven controversial. The barbiturates, chlormethiazole, and recently the safer benzodiazepines transformed the management of these conditions. The benzodiazepines, particularly diazepam and chlordiazepoxide, are now the most used first line agents in the treatment of AWS. In addition, a number of other agents, including baclofen, a GABA-B receptor agonist, have the potential to suppress the alcohol withdrawal syndrome. In this review we review the potential use of baclofen in its role to treat AWS. We summarize initial case reports as well as more recent randomized trials of AWS treatment with baclofen. We conclude that currently there is not enough evidence to support the use of baclofen as a first line treatment for AWS. More research will be needed to determine where baclofen might have a role in second-line management of the Alcohol Withdrawal Syndrome on its own or in combination with benzodiazepines or other agents.

## Introduction

The alcohol withdrawal syndrome, with and without delirium, is challenging with regards to prevention and treatment. For centuries the etiology was unclear and much disputed. In the nineteenth century it was thought that Delirium Tremens (DT) was as a result of extreme intoxication rather than discontinuation of alcohol in habitual drunkards. The patient was thought to have stopped using alcohol as a result of the confusion rather than confusion and delirium being correctly attributed to discontinuation or reduction in the level of drinking. This controversy was eventually resolved by Isbell et al. (1) in their controversial human “guinea pig” study on the etiology of DTs and “Rum Fits.” Such studies also delineated the components of the alcohol withdrawal syndrome without delirium and establish the time course and range of severity of symptoms and signs of the condition. Today severity of the syndrome is measured using one of several rating scales, which also show whether response to treatment is adequate and which guide dosing of medication. One of the most commonly used scales is the Clinical Institute Withdrawal Assessment for Alcohol (CIWA) and its revised forms (2).

In the nineteenth and early twentieth centuries it was also controversial whether alcohol treatment with or without opium was better than “conservative” management without alcohol. Laycock (3) showed that his conservative management with water and beef tea reduced the death rate compared to alcohol and/or laudanum (a tincture of opium). The safety of treatment was again discussed in the twentieth century with the side effect rich use of barbiturates and chlormethiazole until treatment with at least equally effective benzodiazepines became routine.

Whilst benzodiazepines are the drug category of choice, research is still required to investigate other agents in situations of relative contraindication. One such candidate for consideration is baclofen. This drug may have advantages over alternatives, in particular in patients with liver disease or who risk abuse of medication (see below). In this short review we summarize the evidence gathered up to date of baclofen in relation to the management of AWS.

## Alcohol Withdrawal Syndrome

The severity of the alcohol dependence syndrome in patients is a strong predictor of the severity of untreated alcohol withdrawal on sudden cessation of drinking. Those most at risk describe relief drinking, drinking in the morning and throughout the day and some patients top up during the night to maintain a high blood alcohol concentration and avoid uncomfortable withdrawal symptoms. Patients with high alcohol levels but no behavioral signs of intoxication are at definite risk of AWS. The potential for the syndrome is also related to the quantity of alcohol taken daily and the length of continuous drinking. Like alcohol dependence can be mild, moderate or severe so can the withdrawal syndrome. Withdrawal may often be suppressed by a patient by the return to drinking alcohol. This relief of the discomfort of withdrawal is negative reinforcement and perpetuates the dependence syndrome. Medical intervention with safe and effective drugs to relieve this discomfort and reduce the risk of severe complications (seizures, Delirium) is the first stage in recovery from alcohol addiction in motivated patients. Elective detoxification is done at home, in outpatient clinics, day hospitals or specialist residential settings, with the intensity of supervision matching the severity of dependence and the medical risks associated with withdrawal.

However, usually treatment for the alcohol withdrawal syndrome is delivered when cessation of drinking is unplanned and where AWS is the presenting problem or arises in the context of hospitalization for a medical or surgical problem. AWS needs to be considered in all hospital admissions with appropriate screening and early treatment as indicated and dealing with AWS has been an important part of inpatient management in western countries for the last 200 years (4).

The alcohol withdrawal syndrome, with or without delirium, can be fatal if left untreated although the often quoted high rate before modern treatment of up to 35% seems an exaggeration based on either overzealous treatment with non-specific sedatives or a failure to differentiate AWS from comorbid medical conditions as the cause of death. Anticipation of the syndrome reduces the risk and allows for early intervention by pharmaceutical means and reduces withdrawal symptoms during its time-limited course [See (5) for a fuller description].

Symptoms and signs of AWS are result of a hyperexcitable central nervous syndrome. Alcohol enhances the brain's main inhibitory systems via the Gamma-Aminobutyric Acid (GABA)-A receptor for which it is an agonist like benzodiazepines (BZD). In addition, the alcohol's antagonist action on the N-Methyl-D-aspartic acid (NMDA) receptor (one of the 3 glutamate receptors) suppresses the excitatory system leading to the overall CNS depressant effect (It is a curiosity that alcohol was thought of as a “stimulant”). Chronic exposure to alcohol therefore leads to down-regulation of the GABA-A receptors and upregulation of the NMDA receptors. Thus, when alcohol is stopped the balance between GABAergic and glutaminergic systems reverses with decreased inhibition and increased CNS excitation.

The cross-tolerance between alcohol and benzodiazepines, both agonists for the GABA-A/BZD receptor, underpins the effective action of benzodiazepines in suppressing the AWS. Left untreated within a few hours, increased pulse rate, raised blood pressure, tremor, heightened anxiety, and sweating are seen as part of the AWS with autonomic hyperarousal. The risk of seizures is highest in the first 24 h and delirium can appear in up to 5% of cases after 48–72 h. Manifestations are disorientation, confusion, visual, and sometimes auditory hallucinations. Standard medical management of AWS with benzodiazepines and parenteral vitamins is well-established [see (6, 7), and the Cochrane Review on AWS: (8)].

## Background to Baclofen Use in AWS

Baclofen is a specific agonist for the GABA-B receptor. This receptor provides a negative feedback loop for the GABA-ergic system thereby downregulating GABA-A activity and mimicking some of the effects of alcohol induced action on the GABA-A receptor [for more in depth discussion of the baclofen mechanism see (9)]. Baclofen was initially and is commonly used as a muscle relaxant but has been found to have a positive effect on alcohol craving and relapse prevention [for a review see (10)]. In the light of this, animal studies were conducted and baclofen was shown to prevent withdrawal in rats made dependent on alcohol (11, 12). Subsequently, baclofen was successfully used as an open label treatment in humans with AWS (13).

Baclofen is mainly excreted through the kidney and, unlike benzodiazepines, has minimal liver metabolism. Therefore, the risk of toxicity in the numerous patients with AWS and impaired liver function is reduced (14). In many countries there is a rising incidence of alcohol-related liver disease and many patient have undiagnosed cirrhosis and impaired synthetic liver function. However, it is vital to treat AWS at presentation in these patients and it is therefore of benefit to have non-benzodiazepine agents as an alternative to the shorter acting benzodiazepine drugs which are sometimes used in these patients (lorazepam and oxazepam).

In view of baclofen's property to prevent relapse in alcohol dependence, commencement of baclofen during or toward the end of detoxification may be more efficacious than treatment starting after a period of abstinence. The possibility that baclofen is suppressing a “post-withdrawal syndrome” and thereby lessening the likelihood of relapse is also worth studying [For consideration of these broader aspects of baclofen see (15)].

## Methodology

We searched the up to date literature in Medline (on 26/4/18) searching for “baclofen” AND “alcohol withdrawal syndrome” AND “human” to find relevant papers. Seventy six articles were found and were reduced to 51 human clinical papers and those with primary data or an attempt to summarize the evidence were selected. This included studies using baclofen to prevent as well as studies using baclofen to treat established AWS. Our review on baclofen in the management of AWS is separated into open case studies and series, the three available randomized trials and other reviews of the literature.

## Case Studies and Series on Baclofen Use in Alcohol Withdrawal

After it was shown in pre-clinical studies that baclofen prevents withdrawal symptoms in rats (11, 12), a number of open label treatment studies were conducted on in Italy including human studies of baclofen in alcohol withdrawal. In the earliest case series (13) five patients with severe AWS according to CIWA scale (score >20) received open label baclofen (10 mg 8 hourly). Rapid improvement of the CIWA score (Clinical Institute Withdrawal Assessment for Alcohol) was seen on administration of baclofen. This established the possibility of an effect of baclofen on alcohol withdrawal syndrome. An additional single case report by Addolorato et al. (16), showed the drug's efficacy in Delirium Tremens.

These studies are later reviewed in Leggio et al. (17) and in Leggio et al. (18). (See section on reviews). In a review of these studies in 2010, Leggio et al. mention an additional retrospective casenote review of 17 patients treated with baclofen for prevention of AWS. In this chart review at the St. Anthony Hospital in Oklahoma, USA from November 2004 to April 2005 patients were included if they were determined to be at risk for alcohol withdrawal (19). Baclofen was found to suppress the syndrome in 12 out of 14 of the patients (86 %) where AWS hadn't commenced. Of the 3 remaining patients where AWS had commenced there was deemed to be one treatment success and two treatment failures based on pre-determined criteria used (from the diagnostic and statistical manual of mental disorders 4th edition–DSM-IV).

In a similar vein the most recent paper (20) investigates whether prior chronic treatment with “high-dosage” baclofen (range: 60–240 mg) makes a difference during inpatient alcohol detoxification with benzodiazepines in comparison to patients who were not on prior treatment with baclofen. Their study compares 31 patients with chronic pretreatment with high dose baclofen and active alcohol dependency to 31 matched patients with similar alcohol dependence but no prior baclofen treatment. They show that there was no difference in the benzodiazepine dose over a seven day period between the two groups. Within this study there were a small number of patients with cirrhosis−3 in the baclofen group and 5 in the non-baclofen group. They report that there was less need for oxazepam in the 3 patients on baclofen but conclude that it is difficult to generalize the result given these low numbers.

## Drug Trials With Baclofen in Established AWS Utilizing Randomization

Overall, 3 independent trials, one from Italy, one from the US and one from India fulfilled the criteria of randomization, one of which was double blind, the others open label.

In a 2006 study by Addolorato et al. (21), 37 patients with established alcohol withdrawal syndrome were randomly assigned into one of two groups, the first was treated with baclofen 10 mg three times daily, and the second with diazepam calculated according to patient weight. The results showed that baclofen was comparable in efficacy and tolerability to the “gold-standard” diazepam treatment. Outcome measures were calculated using the Clinical Institute Withdrawal Assessment for Alcohol revised (CIWA-Ar) scale of alcohol withdrawal severity, which was also used to guide therapy. Scoring of the withdrawal severity was carried out by members of the research team blinded to the drug under review. All 37 patients who initially enrolled completed the study and the authors report that no rescue protocol medication was required. The median CIWA-Ar score in the baclofen group decreased from over 20 at treatment begin (day 1) to <15 on day 2 and less than 10 on day 3, a comparable decrease to the diazepam group.

The Cochrane review on baclofen in alcohol withdrawal (22) rates the evidence of this study as low quality in view of the absence of blinding participants to the treatment. However, the authors point out that with the exception of slight initial improvements in anxiety in the diazepam group, the two arms performed similarly on outcome measures.

The second study by Lyon (23) enrolled 44 patients with acute symptomatic AWS to their double-blinded trial with 19 randomized to placebo and 25 to baclofen (10 mg three times a day) and patients requiring intravenous benzodiazepines were excluded in this study. Of these 31 completed the 72 h of treatment (13 patients on placebo and 18 on baclofen). Again, in the baclofen group, the CIWA-Ar score decreased from over 12 at treatment begin (d 1) to less than 9 on day 2 to about 8 on day 3. A rescue regimen of lorazepam according to CIWA scores was provided for both groups. The authors report that significantly more rescue lorazepam was required in the placebo group, compared to the baclofen group. CIWA-scores at 8 hour intervals over a five day period show little difference between the two groups which might reflect the practice of lorazepam being given according to CIWA scores.

The Cochrane review (22) also regards this study as low quality, in particular in view of the high attrition rate and it was noted that the study period of 72 hours period was relatively short.

The Indian study by Reddy and Girish (24, 25) sought to compare baclofen with chlordiazepoxide in the context of alcohol detoxification with regards to efficacy and tolerability in uncomplicated alcohol withdrawal. In this open-label trial the authors have included patients with alcohol dependence and alcohol cessation but no established AWS but they do not give specific numbers. Matched groups of 30 participants were randomly assigned either to 30 mg baclofen daily, or 75 mg chlordiazepoxide daily. The CIWA-Ar variant of the CIWA score was used to assess outcome measures during treatment. Lorazepam was available as required for both groups to treat refractory symptoms and there were no drop-outs from this study. Both arms showed a similar reduction in symptoms of alcohol withdrawal, as measured by CIWA-Ar with a mean score of over 23 prior to treatment start (d 1) to <18 on day 2, <15 on day 3, and <10 on day 4. The authors showed that chlordiazepoxide regimen provided a quicker and more effective detoxification. They also state that lorazepam had a larger impact on CIWA-Ar scores in the baclofen group with no significant effect in the chlordiazepoxide group.

Overall, the authors feel that there is a “smoother” detoxification from alcohol with chlordiazepoxide compared to baclofen. This might well be due to the much longer half life of chlordiazepoxide especially when considering its active benzodiazepine metabolite which is in the range of days compared to the half life of baclofen which is in the range of hours (although the pharmacokinetics of these drugs is not very well studied in patients with previous regular alcohol consumption). On measures of symptom-free days and participant satisfaction, the chlordiazepoxide group was preferred.

## Literature Reviews of Baclofen in Alcohol Withdrawal Syndrome

A review in 2008 (17), updated in 2010 (18) reported on open label studies by Italian teams. This included a retrospective casenote review of 17 patients treated with baclofen prophylactically for AWS in the USA. They conclude that the evidence is insufficient to recommend baclofen in AWS treatment. As reasons the open label design, low numbers, attrition bias and detection bias are mentioned but they feel that baclofen is equivalent in efficacy to other drugs and no adverse side effects were reported.

Dixit et al. (26) analyse several drug studies (benzodiazepines and non-benzodiazepines) for the treatment of alcohol withdrawal in the Intensive Care Unit. Baclofen is considered on the basis of one study (23). The small numbers and reduced statistical power but less “breakthrough” lorazepam in the baclofen group was noted.

The Cochrane group uses very strict quality criteria for inclusion in their latest review (22), and included the 3 randomized controlled trials above with 141 participants. They also conclude that insufficient and low quality evidence prevents judgment on efficacy and / or safety of baclofen in AWS.

## Conclusions and Discussion

Overall, despite weaknesses in study design some studies show that baclofen at the dose of 30 mg per day as used in all studies so far may be effective in reducing symptoms of alcohol withdrawal. With just over 140 patients treated for alcohol withdrawal syndrome with baclofen there may be a publication bias with absence of publication of less favorable studies. Also, it is not known whether there would be a benefit in using symptom triggered baclofen doses and allow higher doses. Currently routine treatment with baclofen at a dose of 30 mg per day is not recommended outside trials and benzodiazepines should be used as first line treatment. It is also unclear whether baclofen prevents severe alcohol withdrawal symptoms such as seizures and delirium tremens.

Benzodiazepine use is favored in managing both planned and unplanned AWS, using either a fixed dose or a symptom triggered regime with careful titration for the suppression of signs and symptoms of AWS. Given that tailored and significantly higher doses of baclofen are used in a number of trials in maintenance of abstinence from alcohol, it is surprising that higher (tailored) doses have not been systematically studied for AWS. Usage of higher doses might be more effective but an increased risk of adverse events and baclofen discontinuation syndrome for example on self-discharge would need to be considered (27).

The use of baclofen as a possible adjunct to benzodiazepines in AWS is worthy of further research, in particular to find out whether there are additive or synergistic effects of these two drugs. This is in particular attractive where baclofen maintenance for relapse prevention and treatment of anxiety or post withdrawal symptoms is being considered. The hypothesis that baclofen may offer neuroprotection, possibly alongside acamprosate, in some disease situations, has also been postulated on the basis of animal experiments [e.g., (28)] and it will be worth exploring this in relation to prevention of alcohol related brain damage.

Given that baclofen has a good safety profile in patients with alcohol-related liver disease including cirrhosis and these patients respond to low doses (29) it may also have a potential role in managing AWS in this patient group. It will also be important to further understand the effect of baclofen on different symptoms of the AWS.

In the light of our review we suggest several areas of further research on baclofen in the management of AWS. This includes trial of tailored (higher) baclofen doses in AWS, specific trials in patients with advanced liver disease and in patients with extreme delirium unresponsive to conventional treatment with benzodiazepines. Such studies should be of high quality and preferably multicenter trials.

## Author Contributions

All authors listed have made a substantial, direct and intellectual contribution to the work, and approved it for publication.

### Conflict of Interest Statement

The authors declare that the research was conducted in the absence of any commercial or financial relationships that could be construed as a potential conflict of interest.
